# Characterization and Optimization of Culture Conditions for *Aurantiochytrium* sp. SC145 Isolated from Sand Cay (Son Ca) Island, Vietnam, and Antioxidative and Neuroprotective Activities of Its Polyunsaturated Fatty Acid Mixture

**DOI:** 10.3390/md20120780

**Published:** 2022-12-14

**Authors:** Hoang Thi Minh Hien, Le Thi Thom, Nguyen Cam Ha, Luu Thi Tam, Ngo Thi Hoai Thu, Tru Van Nguyen, Vu Thi Loan, Nguyen Trong Dan, Dang Diem Hong

**Affiliations:** 1 Institute of Biotechnology, Vietnam Academy of Science and Technology, 18 Hoang Quoc Viet Str., Cau Giay, Hanoi 100000, Vietnam; 2 Vietnam Academy of Science and Technology, Graduate University of Science and Technology, 18 Hoang Quoc Viet Str., Cau Giay, Hanoi 100000, Vietnam; 3 Joint Vietnam–Russia Tropical Science and Technology Research Center, 63 Nguyen Van Huyen Str., Cau Giay, Hanoi 100000, Vietnam

**Keywords:** Antioxidant and acetylcholinesterase (AChE) inhibitor, fatty acid in the free form, fatty acid in the alkyl ester form, heterotrophic marine microalga, neuroprotective effect

## Abstract

*Aurantiochytrium* is a heterotrophic marine microalga that has potential industrial applications. The main objectives of this study were to isolate an *Aurantiochytrium* strain from Sand Cay (Son Ca) Island, Vietnam, optimize its culture conditions, determine its nutritional composition, extract polyunsaturated fatty acids (PUFAs) in the free (FFA) and the alkyl ester (FAAE) forms, and evaluate the antioxidation and neuroprotection properties of the PUFAs. *Aurantiochytrium* sp. SC145 can be grown stably under laboratory conditions. Its culture conditions were optimized for a dry cell weight (DCW) of 31.18 g/L, with total lipids comprising 25.29%, proteins 7.93%, carbohydrates 15.21%, and carotenoid at 143.67 µg/L of DCW. The FAAEs and FFAs extracted from *Aurantiochytrium* sp. SC145 were rich in omega 3–6–9 fatty acids (40.73% and 44.00% of total fatty acids, respectively). No acute or subchronic oral toxicity was determined in mice fed with the PUFAs in FFA or FAAE forms at different doses over 90 days. Furthermore, the PUFAs in the FFA or FAAE forms and their main constituents of EPA, DHA, and ALA showed antioxidant and AChE inhibitory properties and neuroprotective activities against damage caused by H_2_O_2_- and amyloid-ß protein fragment 25–35 (Aβ_25-35_)-induced C6 cells. These data suggest that PUFAs extracted from *Aurantiochytrium* sp. SC145 may be a potential therapeutic target for the treatment of neurodegenerative disorders.

## 1. Introduction

As an industry generating billions of dollars annually, microalgal biotechnology is opening up increasing new areas of research. Microalgal biomass is considered a promising source of raw materials for the sustainable production of a wide range of compounds and high value-added products, including functional foods, feed ingredients, animal feed, medicine, cosmetics, bio-stimulants, and bio-actives, as well as for use in agricultural algae extraction, bio-oil production, and biofuel production [1]. Microalgal extracts are ideal sources of biotechnological compounds with high levels of bioactivity, e.g., potent antiviral, anti-microbial, anti-oxidant, anti-inflammatory, anti-cancer, and immune-enhancing properties; neuroprotective activity; and various therapeutic and restorative properties. Furthermore, compounds or metabolites derived from microalgae can be used as antibodies and raw materials for vaccine production against SARS-CoV-2 [2,3].

In recent decades, omega-3 and omega-6 polyunsaturated fatty acids (ω-3 and ω-6 PUFAs, respectively) have received special attention in nutrition and pharmaceutical research and application because of their important roles in human health, including the regulation of biological functions, the prevention and treatment of a variety of diseases related to neurological development [4], improvements in cognition [5], reductions in cardiovascular system stress [6], and anti-inflammatory and anti-cancer actions in humans [1]. ω-3 PUFAs include ALA (linolenic acid; C18:3 ω-3), EPA (eicosapentaenoic acid, C20:5ω-3), and DHA (docosahexaenoic acid, C22:6ω-3), and ω-6 PUFAs include LA (linoleic acid; C18:2 ω-6). All these PUFAs, especially DHA, are produced by the Thraustochytriidae (Thraustochytrids) family, which includes the genera *Schizochytrium, Thraustochytrium, Aurantiochytrium, Labyrinthuloides,* and *Ulkenia*. Studies have been published on the optimal growth conditions and the production of highly effective ω-3 and ω-6 PUFA strains belonging to these genera [7,8,9]. In 2001, the US FDA affirmed that DHA and ARA oils derived from microalgae are generally recognized as safe (GRAS) for use in infant formula. Omega-3 products derived from heterotrophic marine microalgae and prepared for human consumption include Life’sDHA^®^, Life’s™OMEGA, DHASCO (from *Schizochytrium* sp. microalgae), and DHASCO-T (from *Crypthecodinium cohnii*) marketed by DSM and the Martek Biosciences Corporation of Columbia, USA. Moreover, mixtures of PUFAs extracted from algae have been added to commercial products such as yogurt, confectionery, and beverages [10]. Falk et al. [11] evaluated the toxicity of DHA-rich oils from *Schizochytrium* sp. (DHA accounted for 41.37% the total fatty acids—TFAs) and showed using a rat model that this oil was safe for the embryo or fetus and during growth. Omega-3 fatty acids can help improve factors that cause nerve cell damage, are able to protect cerebral blood vessels, and can increase metabolism and brain activity in mice with Alzheimer’s disease [12]. Several companies including Algae, Parry Nutraceuticals, Fermentalg, Algaetech International, AlgaeBiotech, Algae to Omega Holdings, and Alltech Algae are performing nutraceutical research and conducting product development based on microalgal oils that are rich in EPA and DHA. PureOne^TM^ is an EPA- and DHA-rich algal oil supplement sold on the market as capsules [13].

Various methods can be used for the extraction and purification of PUFAs, such as the use of urea complexes, low-temperature solvent crystallization, fractional distillation, hydrolysis, enzymatic techniques, and high-pressure liquid chromatography (HPLC) [14,15]. Of the above methods, the use of urea complexes is the most suitable for attaining PUFA-rich samples [15].

Today, along with increases in life expectancy and changes of population demographics, neurodegenerative diseases such as Alzheimer’s and Parkinson’s disease (PD) are becoming seriously prevalent [16,17,18]. The marine environment is known to be a rich source of chemical structures that have many beneficial biological effects on human health. It is widely accepted that natural marine products have many unusual and unique chemical properties. Around the world, based on molecular models and chemosynthetic methods, new drugs with greater efficacy and specificity for the treatment of human diseases have been developed [19,20]. Among marine organisms, heterotrophic marine microalgae have been the specific targets of numerous studies that have shown their potential as rich sources of structurally diverse and biologically active compounds with great pharmaceutical and biomedical potential, including PUFAs [1,2,16,17,21,22,23]. Correlative epidemiological studies suggest that the high consumption of food rich in ω-3 PUFA is associated with better health and possibly the prevention of age-related cognitive impairment or Alzheimer’s disease (AD). Many longitudinal or case–control studies have shown an association between ω-3 PUFA consumption or blood levels and lower risks of dementia or AD [24,25,26]. Recently, a cross-sectional study in a Finnish cohort reported that higher serum concentrations of long-chain ω-3 PUFA were associated with better performance in neuropsychological tests [27]. Preclinical studies involving controlled diets have consistently shown that increasing DHA concentrations in the brain improves rodent performance in a wealth of different memory tests [28]. This has been confirmed in various animal models of AD-like neuropathology. DHA-induced decreases have been reported in amyloid levels, neurofibrillary tangles (Tau), or synaptic neuropathologies in animal models of AD [28,29,30]. More specifically, lower amyloid beta protein (Aβ) levels in the brains of amyloid protein precursor (APP) transgenic mice after high DHA intake have been reported by at least four groups [31,32,33,34]. Other evidence suggests that DHA may also act more directly on neuronal function by progressively integrating cell membranes without necessarily targeting AD neuropathology [29]. A reduction in markers of neuroinflammation has also been observed following n-3 PUFA intake, which could contribute to a therapeutic effect in neurodegenerative diseases [35].

*Aurantiochytrium* is an oleaginous microorganism genus in the Thraustochytriaceae family that has attracted attention because of its ability to produce high levels of PUFAs and other compounds such as squalene, astaxanthin, and exopolysaccharide. A few reports have demonstrated that ethanol extracts obtained from *Aurantiochytrium* sp. have anti-inflammatory properties [36,37]. However, knowledge is limited on the effects of *Aurantiochytrium* sp. and its bioactive compounds on neuroprotection. Thus, the main aims of the present study were to isolate *Aurantiochytrium* sp. SC145 from decomposing leaves and seagrass collected from Sand Cay (Son Ca) Island, Vietnam, and to optimize its culture conditions. Then, PUFAs in the forms of alkyl esters (FAAE) and free fatty acids (FFA) were extracted, assessed regarding their acute and subchronic toxicity in rats, and evaluated in terms of their antioxidation and neuroprotective activities.

## 2. Results and Discussion

### 2.1. Morphology and Characterization of Aurantiochytrium sp. SC145

**Figure 1:**
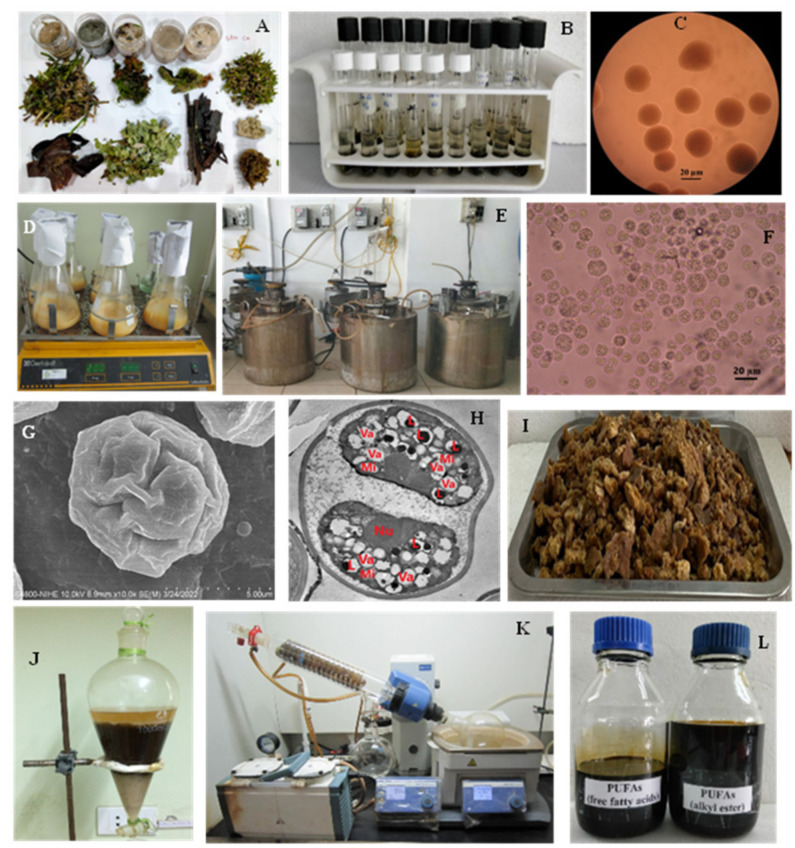
Some images of isolated samples from Sand Clay (Son Ca) Island. (**A**) Collected specimens; (**B**) test tubes containing specimens; (**C**) colony morphology under light microscope; (**D**) SC145 strain cultured in a 1 L flask; (**E**) SC145 strain cultured in a 30 L fermentor. Cell morphology observed with (**F**) a microscope at a magnification of 1200×; (**G**) with a scanning electron microscope (SEM) at a magnification of 20.000×; (**G**) with a transmission electron microscope (TEM) at a magnification of 20.000×. (**H**) Nu: nucleus; Va: vacuole; L: lipid; Mi: mitochondria. (**I**) Dry cell weight; (**J**–**L**) extraction of PUFAs in the alkyl ester (FAAE) and free fatty acid (FFA) forms, respectively.

From samples collected in May–June 2021 near the coast of Sand Cay (Son Ca) island, Vietnam, (Figure 1A), we successfully isolated (Figure 1B,C) and stably cultured *Aurantiochytrium* sp. SC145 under laboratory conditions (Figure 1D,E).

**Figure 2:**
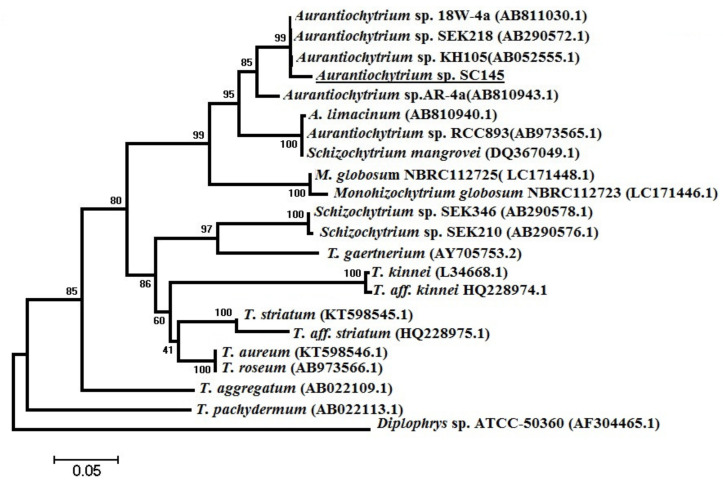
Neighbor-joining phylogenetic tree of *Aurantiochytrium* sp. SC145 based on 18S rRNA.

Additionally, a sequence of a partial 18S rRNA gene of the SC145 strain was determined. Phylogenetic analysis based on the 18S rRNA gene revealed that the SC145 strain belonged to the *Aurantiochytrium* genus. The phylogenetic tree indicated that the nearest phylogenetic neighbor of the isolate of SC145 was the *Aurantiochytrium* sp. KH 105 strain (accession number AB052555) with a similarity coefficient of 99.4% (Figure 2). The GenBank accession number for the 18S rRNA gene sequences of *Aurantiochytrium* sp. SC145 is ON597428.

At species level, based on the morphological characteristics observed under light microscopy (Figure 1F), SEM (Figure 1G), and TEM (Figure 1H), as well as the sequence of the partial 18S rRNA gene, we concluded that the SC145 sample belonged to *Aurantiochytrium* sp. SC145 (Figure 1 and Figure 2).

### 2.2. Optimization of Culture Conditions

#### 2.2.1. Cultivation of *Aurantiochytrium* sp. SC145 in an Erlenmeyer flask

The effects of environmental parameters on the cell density, dry cell weight (DCW), and lipid content of the *Aurantiochytrium* sp. SC145 strain were determined (Table 1).

*Effect of the nutritional media*. The *Aurantiochytrium* sp. SC145 strain grew well in three forms of culture media (M1, Bajpai, and GPY) (Table 1). In particular, the M1 medium showed the highest cell density, DCW, and lipid content of 120.17 ± 2.76 × 10^6^ cells/mL, 12.27 ± 0.53 g/L, and 27.41 ± 0.73% DCW, respectively, and differences were statistically significant between the experimental formulas (*p* < 0.05) after 120 h of culture. Therefore, M1 was selected as a suitable medium for the growth of the SC145 strain.

*Effect of incubation temperature.* Temperature has been shown to have maintenance effects on the normal state of microalgal membrane lipids [38]. Table 1 shows that the highest cell density, DCW, and lipid content of 123.68 ± 2.73 × 10^6^ cells/mL, 12.39 ± 1.62 g/L, and 27.82 ± 1.67% DCW, respectively, were reached at a temperature of 28 °C, followed by the respective values attained at 25 °C. There were no statistically significant differences (*p* > 0.05) between the temperatures of 25 and 28 °C after 120 h of culture. The results obtained in this study were consistent with those of Russo et al. [38], where the *A. mangrovei* strain RCC893 showed its highest growth and lipid content at 28 °C. Meanwhile, algae cells can hardly grow at temperatures of above 32 °C or below 20 °C, so the biomass and lipid content significantly decreased accordingly. Therefore, a suitable temperature for the growth of the SC145 strain was set to 25–28 °C.

*Effect of carbon sources.* Carbon sources affect the regulation of cell metabolism and fatty acid synthesis in microalgae [39]. The results presented in Table 1 show that the highest cell density, DCW, and total lipid values of 121.48 ± 1.06 × 10^6^ cells/mL, 12.79 ± 1.15 g/L, and 26.63 ± 1.18% DCW, respectively, were reached after 120 h of culture when using glucose as a carbon source. When algae were cultured in the medium with glucose, algae cells were uniform in size and their lipid bodies were rounder and darker compared with those cultured with other carbon sources. The differences between carbon sources were statistically significant (*p* < 0.05), because glucose accelerates growth and increases the metabolism and fatty acid synthesis of *Aurantiochytrium* sp. The highest specific growth rate of *Aurantiochytrium limacinum* ATCC MYA-1381 (0.21/h) was obtained previously using glucose as the carbon source [39]. Therefore, glucose was used as the carbon source for our further studies.

*Effect of glucose concentration.* The results in Table 1 show that at a 3% glucose concentration, the SC145 strain had the highest cell density, DCW, and lipid content of 127.62 ± 2.03 × 10^6^ cells/mL, 12.69 ± 1.12 g/L, and 26.76 ± 0.24% DCW, respectively, after 120 h of culture. However, differences between glucose concentrations of 3%, 5%, 7%, and 9% were not statistically significant (*p* > 0.05). Therefore, a glucose concentration of 3% was used for further experiments.

*Effect of nitrogen sources.* Nitrogen also plays an important role in the growth and development of microalgae including lipid production [40]. The results in Table 1 show that the cell density, DCW, and total lipid content of the SC145 strain reached their highest values of 125.48 ± 1.89 × 10^6^ cells/mL, 12.36 ± 0.75 g/L, and 28.32 ± 1.54% DCW, respectively, after 120 h of culture using a yeast extract as the nitrogen source. In the work of Heggeset et al. [41], a yeast extract was used as a source of growth factors and was shown to be effective for the regulation of the fatty acid accumulation of *Aurantiochytrium* sp. T66. In addition to yeast extracts, the SC145 strain can also use NaNO_3_ as a nitrogen source for growth. However, the lipid content was found to be significantly higher when using a yeast extract than when using NaNO_3_ (*p* < 0.05). Therefore, a yeast extract was selected as the nitrogen source for the growth of this strain in further experiments.

*Effect of yeast extract concentrations.* The results in Table 1 show that the DCW and lipid content increased linearly as the yeast extract concentration increased from 0.5 to 1.5%. The highest cell density, DCW, and lipid content of 128.47 ± 1.08 × 10^6^ cells/mL, 12.85 ± 1.37 g/L, and 28.34 ± 1.06% DCW, respectively, were reached after 120 h of culture at a yeast extract concentration of 1%. However, there were no statistically significant differences (*p* > 0.05) in the DCW and lipid content in the medium when using 1% and 1.5% yeast extracts. Therefore, in order to reduce production costs, a yeast extract concentration of 1% was selected for the cultivation of the SC145 strain on the fermentor scale.

In summary, the *Aurantiochytrium* sp. SC145 strain showed the best growth rate in the M1 medium (3% glucose and 1% yeast extract at a temperature of 25–28 °C) with the highest cell density, dry biomass, and lipid content of 128.47 ± 1.08 × 10^6^ cells/mL, 12.85 ± 1.37 g/L, and 28.34 ± 1.06%, respectively, achieved after 120 h of culture.

#### 2.2.2. Cultivation of *Aurantiochytrium* sp. SC145 in a 30 L fermentor

According to Chang et al. [42], a glucose carbon source is required for the development of thraustochytrids, and different genera have different glucose utilization requirements for growth. Therefore, at a fermentation scale of 30 L, the effects of glucose concentrations and nitrogen sources of a 1% pure yeast extract (foreign yeast extract, Merk, Germany) and a 1% industrial yeast extract (produced by the Institute of Food Industry, Vietnam) were studied, with the aim of reducing production costs for large-scale cultivation.

*Effect of glucose concentrations on the growth of Aurantiochytrium sp. SC145 in a 30 L bioreactor.* The effects of investigated glucose concentrations (3, 6, 9, and 12% glucose) on the growth of *Aurantiochytrium* sp. SC145 are shown in Table 2. The results showed that in the 30 L fermentor, *Aurantiochytrium* sp. SC145 grew well at all glucose concentrations. The biomass of this strain rapidly increased within 24 h and reached its maximum after 120 h of culture. After increasing culture time to 168 h, the cell growth tended to decrease.

The results in Table 2 show that *Aurantiochytrium* sp. SC145 reached its maximum cell density, DCW, and lipid content of 154.75 ± 1.14 × 10^6^ cells/mL, 31.15 ± 1.13 g/L, and 24.92 ± 0.31% DCW, respectively, at 9% glucose concentration after 120 h of culture. The growth was similar at 12% glucose concentration, and there was no statistically significant difference in growth at glucose concentrations of 9 and 12% (*p* > 0.05). Therefore, for the cultivation of *Aurantiochytrium* sp. SC145 in a 30-L fermentor, a glucose concentration of 9% should be selected in order to increase algae growth and save costs.

*Effects of pure yeast extract and industrial yeast extract (homemade) on the growth of Aurantiochytrium sp. SC145 in a 30 L fermentor*. Changes in the DCW and lipid content of *Aurantiochytrium* sp. SC145 in a 30 L fermentor when using pure and industrial yeast extracts as the nitrogen sources are shown in Table 2. The results show that after 120 h of culture there were no differences in cell density, DCW, and lipid content when the studied strain was cultured with pure yeast or industrial yeast extract as the nitrogen source. The cell density, DCW, and lipid content of *Aurantiochytrium* sp. SC145 reached 145.27 ± 1.83 × 10^6^ cells/mL, 31.89 ± 1.17 g/L, and 26.15 ± 1.56% DCW, respectively, with the pure yeast extract and 148.51 ± 0.67 × 10^6^ cells/mL,31.18 ± 2.63 g/L, and 25.29 ± 1.43% DCW, respectively, with the homemade industrial yeast extract. Therefore, we decided to use the industrial yeast extract to grow the biomass for the subsequent experiments.

In summation, in the 30 L fermentor, *Aurantiochytrium* sp. SC145 grew best with a glucose concentration of 9% and an industrial yeast extract concentration of 1%, reaching the highest cell density, dry biomass, and lipid content values of 148.51 ± 0.67 × 10^6^ cells/mL, 31.18 ± 2.63 g/L, and 25.29 ± 1.43% DCW, respectively.

The conditions for the biomass cultivation of heterotrophic marine microalga *Aurantiochytrium* sp. SC145 as the raw material for PUFA extraction were an M1 medium containing 3% glucose and 1% yeast extract, at a temperature of 25–28 °C for the flask (Figure 1D) and an M12 medium containing 9% glucose and 1% industrial yeast extract on the 30 L fermentor scale (Figure 1E). In the 30 L fermentor, the DCW (Figure 1I) and lipid, protein, and carbohydrate contents reached their highest values of 31.18 ± 2.63 g/L, 25.29 ± 1.43%; 7.93 ± 0.17%, and 15.21 ± 0.02% of DCW, respectively. In addition, the carotenoid and astaxanthin contents reached 143.67 ± 0.35 and 8.07 ± 0.17 µg/L, respectively (Table 3). The fatty acids mainly comprised omega 3–6–9 fatty acids, which accounted for 51.60% of TFA (Table 4). These fatty acids act as independent lipids, but they can interact with other biomolecules in the body to maintain homeostasis and ensure good health in humans. Simopoulos [43] suggested that human beings evolved on a diet with a healthy omega-6 to omega-3 fatty acid ratio of 4:1–5:1, and this ratio should not exceed 10:1. In addition, research has indicated that a ratio of 25:1 favorably modified the erythrocyte fatty acid profile of spontaneously hypertensive rats [44].

The fatty acid composition of the biomass of the SC145 strain cultured in the 30 L fermentor after 120 h of cultivation is shown in Table 4. The results indicate that the fatty acid composition mainly comprised palmitic acid (19.40% of TFA), oleic acid (32.10% of TFA), LA (0.20% of TFA), ALA (16.20% of TFA), EPA (0.80% of TFA), and DHA (1.90% of TFA). The fatty acid composition mainly included omega 3–6–9 fatty acids, which accounted for 51.70% of TFA; i.e., omega-3, omega-6, and omega-9 fatty acids accounted for 18.90% of TFA, 0.40% of TFA, and 32.40% of TFA, respectively. The main omega-9 fatty acid in the brain is oleic acid (18:1 ω-9)—an important fatty acid for the brain and the cardiovascular system that can be synthesized or obtained from food [45]. According to Johnson and Bradford [46], omega-3 fatty acids are often associated with the initiation of anti-inflammatory responses, while omega-6 fatty acids are associated with proinflammatory responses. Omega-9 fatty acids serve as essential components for other metabolic pathways. These fatty acids act as independent lipids, but they can interact with other biomolecules in the body to maintain homeostasis and ensure good health in humans.

### 2.3. Extraction of PUFAs Rich in Omega 3–6–9 Fatty Acids in the FFA Form

**Figure 3:**
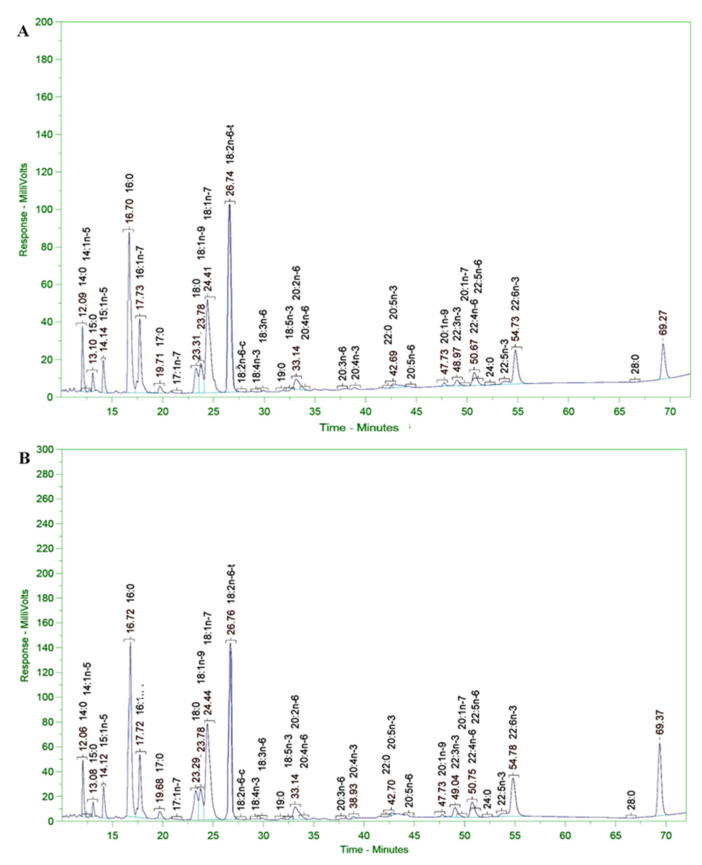
Chromatography of fatty acid composition of PUFAs in the FFA (**A**) and FAAE forms (**B**).

We extracted PUFAs in the free form containing omega 3–6–9 fatty acids (Figure 1J–L) from the biomass of the SC145 strain. Here, the lipid extraction conditions were optimized with the use of an n-hexane solvent, a reaction temperature of 70–75 °C, an extraction time of 3 h, and continuous stirring, which yielded the highest lipid extraction efficiency of 28% of DCW. The saponification reaction conditions were 7% concentration of NaOH in ethanol, a reaction temperature of 70–75 °C, a reaction time of 3 h, and continuous stirring, yielding the highest content of free fatty acids of 90% of lipids. Finally, the conditions for the complexation of the free fatty acid mixture with urea were optimized as follows: the free fatty acid–urea ratio was 1:4 (*v*:*w*), the urea–ethanol ratio was 1:9 (*w*:*v*), and the crystallization temperature was 4 °C. The efficiency of the extraction process of PUFAs in the FFA form reached about 23–25% of crude oil (lipids) and 17–18% of DCW. The analysis results of fatty acid composition in the PUFAs mixture after the extraction process are shown in Table 5 and Figure 3A. The obtained results in Table 5 show that the total content of omega 3–6–9 fatty acids reached 40.73 ± 0.19% of TFA, i.e., the omega-3 content was 24.50 ± 0.05% of TFA, the omega-6 content was 11.20 ± 0.10, the omega-9 content was 5.00 ± 0.03, and the DHA content was 7.36 ± 0.03% of TFA.

### 2.4. Extraction of PUFAs Rich in Omega 3–6–9 Fatty Acids in the FAAE Form

PUFAs containing omega-3, -6, and -9 fatty acids in the FAAE form were extracted from the biomass of the SC145 strain (Figure 1J–L). The appropriate conditions for TFA extraction from the biomass of the SC145 strain were: an HCl catalyst; a solvent–biomass ratio of 10:1 (*v*:*w*), and a temperature of 70–75 °C for 3 h. The TFA extraction efficiency was about 42–43% of DCW. Finally, the conditions for the enrichment of a PUFA blend rich in omega 3–6–9 fatty acids in the FAAE form comprised a TFA–urea ratio of 1:4 (*w*:*w*), a urea–methanol ratio of 1:5 (*w*:*v*), and a crystallization temperature maintained at 4 °C. The efficiency of PUFA extraction in the FAAE form was about 12–13% of DCW. The results of fatty acid analysis of the PUFA mixture after extraction are shown in Table 5 and Figure 3B. Table 5 shows that the total content of omega 3–6–9 fatty acids reached 44.00 ± 0.21% of TFA, i.e., the omega-3 content was 25.66 ± 0.06% of TFA, the omega-6 content was 13.76 ± 0.13 of TFA, the omega-9 content was 4.56 ± 0.02% of TFA, and the DHA content was 8.99 ± 0.03% of TFA. These fatty acids have the potential to enhance mitochondrial fatty acid oxidation and antioxidant capacity in human atrial myocardia [47,48].

Omega-3 fatty acids have potent biological effects on lipoprotein metabolism, platelet function, cytokine production, coagulation, fibrinolysis, and inflammatory factors. Omega-3 fatty acids increase the antioxidant capacity, strengthen the defense system, and may prevent diabetic complications in patients with type 2 diabetes [49]. Therefore, fatty acids in the FFA and FAAE forms were used for the experiments on the neuroprotective effects of these PUFA mixtures.

### 2.5. Acute Toxicity Test

No signs of toxicity were observed in the FAAE and FFA PUFAs over 72 h or after 7 days of acute toxicological evaluation. The lethal doses of the PUFAs in the FAAE and FFA forms were not determined; it might be suggested that PUFAs isolated from *Aurantiochytrium* sp. SC145 in the FAAE or FFA forms are not toxic to mice. Our findings are in line with reports by Falk et al. [11] and Thom and Hong [15]; Falk et al. [11] reported that DHA-rich oil from *Schizochytrium* sp. (DHA accounted for 41.37% of TFAs) at a dose of 5000 mg/kg/day was safe for embryos and fetuses and during the growth of mice, and Thom and Hong [15] reported that bio-oil rich in omega 3–6 fatty acids (DHA, EPA, and DPA) was safe and had the ability to improve memory and learning ability in mice.

### 2.6. Subchronic Toxicity Test

#### 2.6.1. Behavioral Observations and Body Weight Trends

All mice in the control and treatment groups showed the same appearance and behaviors regarding activity, eating, hair, skin, mucosa, and secretions throughout the observation period. No mortalities were detected in either of the groups during the study period. The body weights of the mice in all five studied groups progressively increased over 90 days with the administration of the PUFAs in the FAAE and FFA forms. After 90 days, there were no significant differences in changes of body weight among groups (Table 6). The PUFAs in the FAAE or FFA forms at different doses did not affect the development of body weight in the studied mice.

#### 2.6.2. Hematological and Biochemical Parameters

The hematopoietic system is very sensitive to toxic substances. Hematological studies are important indicators of the pathophysiological status of humans and animals [50]. For example, an increase in the count of white blood cells (WBCs), which are synthesized in adult bone marrow and fetal hematopoietic organs, indicates the strengthening of an organism’s defense system [50]. The number of free phagocytes can quantify the number of infection-fighting WBCs in the blood. High WBC counts may be associated with cancer, microbial infections, and other diseases [50]. Red blood cells (RBCs) contain oxygen-carrying hemoproteins, and RBC tests can diagnose anemia and other related conditions. Leukocytes include basophils, eosinophils, lymphocytes, monocytes, and neutrophils, and their numbers increase in response to infections and allergic reactions [50]. Here, there were no significant differences after 90 days in any of the hematological parameters (RBC, hemoglobin, hematocrit, average volume of erythrocytes, WBC, and platelet count) between mice treated with the PUFAs in the FAAE or FFA forms and mice in the control group (Table 7).

Biochemical parameters were evaluated in order to determine whether the supplementation of PUFAs in the FAAE or FFA forms could change liver or kidney function in mice. Liver and kidney function analyses can be employed to identify the toxicity of drugs, compounds, and plant extracts [50]. Aspartate aminotransferase (AST) and alanine aminotransferase (ALT) are liver enzymes, and their upregulation indicates liver injury. Kidney function can be assessed using creatinine analysis [50]. In this study, no significant changes were observed in ALT, AST, nor creatinine in mice at all oral doses of PUFAs in the FAAE or FFA forms compared with controls (Table 7). Therefore, the oral administration of PUFAs in the FAAE and FFA forms did not affect hepatocyte and renal function in mice.

Reductions in albumin also affect liver function. As the liver is a major site of protein synthesis, any decrease in its function may be indicative of hepatocyte damage. Impaired hepatocellular function may result in reduced concentrations of serum albumin. Albumin prevents fluids from leaking out of blood vessels, and low albumin levels may also be characteristic of infection [51]. Our data revealed that the dose of PUFAs in the FAAE or FFA form did not significantly influence the content of serum albumin in mice. Therefore, none of the doses of the tested FAAE and FFA forms damaged hepatocellular function. Clinical trials previously showed that ω-3 PUFA consumption was inversely correlated with incidence of coronary heart disease (CHD). The relationship between dietary ω-3 PUFA and CHD is believed to be only partially mediated by their effects on plasma lipoprotein profiles. Omega-3 PUFAs have been shown only slightly to reduce total and low-density lipoprotein (LDL) cholesterol, probably because they crowd saturated fatty acids out of the diet. Data on high-density lipoprotein (HDL) cholesterol suggest that ω-3 PUFAs produce only a small increase in this fraction. The effect of omega-3 PUFA supplementation on plasma triglycerides (TGs) was found to be much more important, with a reduction of about 25% in normolipidemic subjects and about 50% in hypertriglyceridemia patients [52]. Here, mice treated with PUFAs in the FAAE or FFA form at a dose 353.3 mg/kg/day showed slightly decreased total cholesterol content compared with the control group (Table 7). However, changes were not significant between the experimental groups (1, 2 3, and 4) and the control.

Our obtained results were in agreement with a report on certain commercial DSM nutritional products including DHASCOTM (oil extracted from *Crypthecodinium*) and DHASCO^®^-S (oil extracted from *Schizochytrium* sp.) [10], in which products with a DHA content of 350 mg/g oil were tested for safety in mice at a dosage of 4000 mg/kg body weight/day, and DHASCO-B (oil extracted from *Schizochytrium* sp.) was tested at a dosage of 4260 mg/kg body weight/day [10]. The results showed that the products were generally safe with no toxicity.

### 2.7. Scavenging Activity of DPPH Radical

Radical scavenging activities play an important role in the prevention of free radical-induced cell damage. DPPH free radical scavenging has been successfully applied to screen the antioxidant activity of extracts and other substances from natural products [53]. In this study, the PUFAs in the FAAE and FFA forms showed radical scavenging activity (Table 8). The IC_50_ values of the PUFAs in the FAAE and FFA forms were 294.98 ± 4.03 μg/mL and 239.21 ± 5.01 μg/mL, respectively. In our study compared with others, PUFAs in the FAAE form from *Aurantiochytrium* sp. SC145 showed less activity than PUFAs in the FAAE form extracted from soybeans and sunflowers reported by Pinto et al. [54], but higher activity than the EPA/DHA omega-3 fatty acids reported by Kotue et al. [48]. It has been suggested that various saturated and unsaturated fatty acids that are present in soybeans, palms, coconuts, corn, sunflowers, and many other food products show high antioxidant activity [55]. According to our determination of the fatty acid composition in PUFAs in the FAAE and FFA forms from *Aurantiochytrium* sp. SC145, ALA (20.13 and 22.02%, respectively) was the dominant unsaturated fatty acid, followed by DHA (7.36 and 8.99%, respectively), LA (5.63 and 5.12%, respectively), and EPA (1.40 and 0.36%, respectively) (Table 5 and Figure 3). Those PUFAs, except for LA, showed good antioxidant activity. The IC_50_ values were 262.54 ± 5.47 μM for ALA, 197.87 ± 3.63 μM for EPA, and 285.04 ± 2.71 μM for DHA (Table 8). Taken together, these results suggest that EPA, DHA, and ALA account for the antioxidant activity of PUFAs in the FAAE and FFA forms.

### 2.8. AChE Inhibitory Activity

PUFAs play essential roles in neuroprotective and anti-inflammatory activity [56]. Evidence suggests that PUFAs can inhibit increases in apoptosis and prevent reductions in neurogenesis [57,58]. The enzyme AChE is involved in accelerated amyloid formation, which is considered to be one cause of AD progression [59]. Therefore, the inhibition of AChE not only plays a key role in reducing the aggregation of β-amyloids but also enhances cholinergic neurotransmission in the brain. The AChE inhibitory activities of PUFAs in the FAAE and FFA forms, LA, ALA, EPA, and DHA are presented in Table 8. All tested samples except for LA showed AChE inhibitory activity (Table 8). The IC_50_ values of the PUFAs in the FAAE and FFA forms, ALA, EPA, and DHA were 2.65 ± 0.16 μg/L, 4.9 ± 0.65 μg/L, 10.80 ± 0.66 μM, 5.03 ± 0.27 μM, and 4.40 ± 0.31 μM, respectively. According to the obtained results, we suggest that unsaturated fatty acids such as ALA, EPA, and DHA are the main components responsible for the AChE inhibitory activities of PUFAs in the FAAE and FFA forms. PUFAs in the FAAE and FFA forms could be used as potential anti-amyloidogenic drugs for treatment of Alzheimer’s disease.

### 2.9. Cytotoxicity Effect of Polyunsaturated Fatty Acids on C6 cells

**Figure 4:**
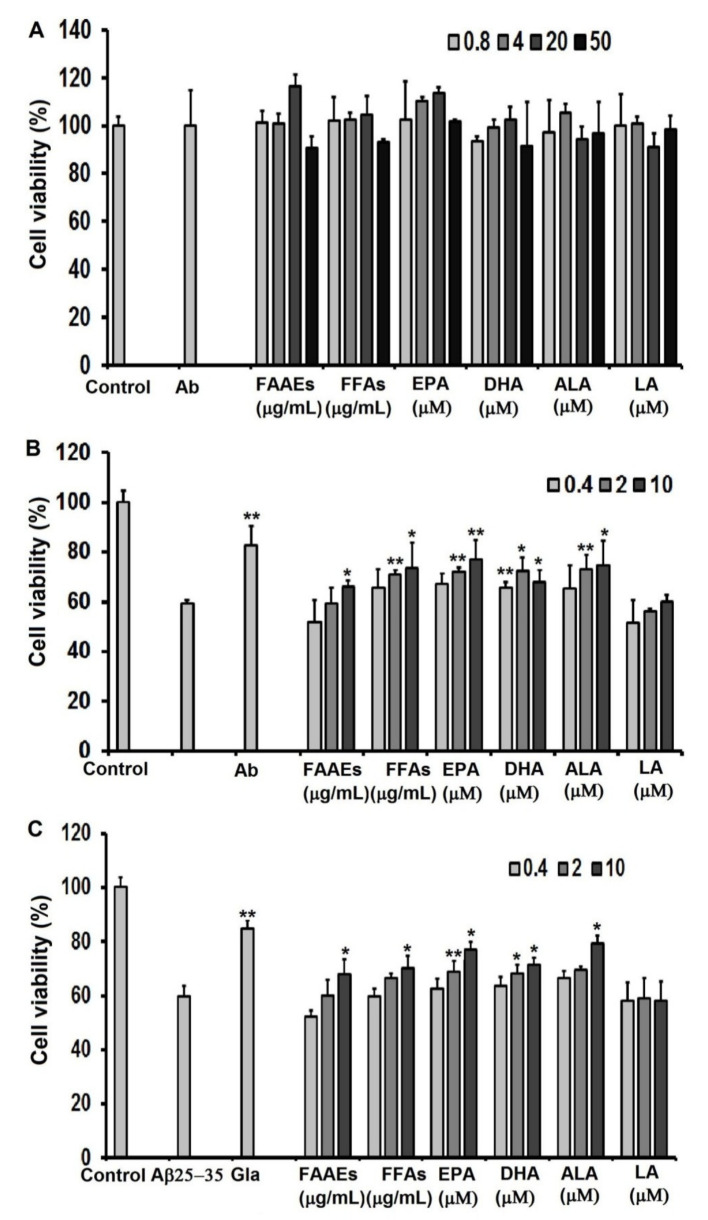
Effects of PUFAs in the FAAE and FFA forms and EPA, DHA, ALA, and LA from *Aurantiochytrium* sp. SC145 on (**A**) the cell survival of C6 cells, and their neuroprotective activities against (**B**) H_2_O_2_- and (**C**) Aβ_25-35_-induced neurotoxic in C6 cells. Ab: ascorbic acid at a concentration of 20 μg/mL. The data are expressed as mean ± SEM (*n* = 3). Significant differences in the cell damage induced by H_2_O_2_ in Figure (**B**) and the Aβ_25-35_ group in Figure (**C**) are denoted by: * *p* < 0.05, ** *p* < 0.001.

The effects of PUFAs in the FAAE and FFA forms, EPA, DHA, ALA, and LA at different concentrations on the survival ability of C6 cells were tested (Figure 4A). The obtained results showed that C6 cells had a survival rate of 98% when incubated with PUFAs in the FAAE or FFA form at concentrations of 0.4, 2, 10, and 50 µg/mL and EPA, DHA, ALA, and LA at concentrations of 0.4, 2, 10, and 50 µM over 24 h (Figure 4A). These results suggested that all tested compounds had no cytotoxic effect on the C6 cell line at maximum concentrations of 50 µg/mL for PUFAs in the FAAE and FFA forms or 50 µM for EPA, DHA, ALA, and LA.

### 2.10. Neuroprotective Effects of PUFAs against Damage Caused by Oxidative Stress Induced by H_2_O_2_ on C6 cells

Oxidative stress is considered an important factor in numerous diseases including cardiovascular disease, chronic obstructive pulmonary disease, and neurodegenerative disease [60]. Research has shown that directly adding H_2_O_2_ to a culture medium can lead to oxidative stress and the death of cells [61]. Therefore, in this study, we assessed the cytoprotective effect of PUFAs in the FAAE and FFA forms and EPA, DHA, ALA, and LA against oxidative damage from oxidative stress caused by H_2_O_2_ in C6 cells (Figure 4B).

The results in Figure 4B show that treatment with 10 μM of H_2_O_2_ significantly decreased cell viability by 59.2% and that this rate reached 100% when the cells were cultured in a medium without H_2_O_2_ (the control). Incubating the cells with ascorbic acid before adding H_2_O_2_ significantly inhibited the damaging effect of H_2_O_2_ on C6 cells. Levels of viability in cells pretreated with PUFAs in the FAAE and FFA forms at concentrations of 10 μg/mL were increased by 12.63 and 24.30%, respectively, compared with cells treated only with H_2_O_2_. PUFAs rich in omega 3–6–9 fatty acids in the FFA form were more active those in the FAAE form. Although the FFA form has higher neuroprotective activity than fatty acids in the FAAE form, the FFA form is often unstable because it is much more susceptible to oxidation than the FAAE form. Therefore, in practice, it is common to produce oils rich in fatty acids for large-scale commercialization; the fatty acids often have to be produced in the form of alkyl esters.

It was noted that pretreatment with EPA and ALA at concentrations of 2 μM or 10 μM and DHA at 0.4 μM, 2 μM, or 10 μM significantly ameliorated the neurotoxic effect of H_2_O_2_ compared with the H_2_O_2_ group.

### 2.11. PUFAs in the FAAE and FFA Forms Protect C6 Cell Line against Aβ_25-35_-Induced Cytotoxicity

In patients with AD, Aβ_25-35_ in the brain aggregates into clumps called oligomers that can accumulate and form deposits called amyloid plaques, which are thought to be a major pathologic mechanism of AD. In various studies, Aβ_25-35_-induced neurotoxicity has been attributed to Ca^2+^ influx, the generation of reactive oxygen species (ROS), the induction of apoptosis, and other causes. The results of the present study confirm that Aβ_25-35_ at a concentration of 20 μM can cause neural cell death, accounting for about 42.17% cell death compared with untreated cells. The pretreatment of C6 cells with FAAE and FFA or galantamine (0.1 μg/mL) prior to the addition of Aβ_25-35_ significantly ameliorated the neurotoxic effect of Aβ_25-35_ compared with the Aβ_25-35_ group (Figure 4C). The administration of PUFAs in the FAAE and FFA forms at a concentration of 10 μg/mL led to significant increases of 13.31% and 17.14%, respectively, in the cell survival rate. It was also found that EPA, DHA, and ALA protected C6 cells against Aβ_25-35_-induced cytotoxicity. When cells were pre-incubated with 10 μM of EPA, DHA, and ALA, cell viability was 77.12%, 71.44%, and 79.10%, respectively, compared with the Aβ_25-35_ treatment group. These results indicated that the FAAE and FFA PUFAs derived from *Aurantiochytrium* sp. SC145 (specifically the predominating unsaturated fatty acids of EPA, DHA, and ALA) had protective effects against Aβ_25-35_-induced cytotoxicity in C6 cells.

These data suggest that PUFAs rich in omega 3–6–9 fatty acids from *Aurantiochytrium* sp. SC145 have neuroprotective effects against H_2_O_2_ and Aβ_25-35_-induced neurotoxicity in C6 cells due to their antioxidant and AChE inhibitory activities.

Mixtures of fatty acids in both the alkyl ester and free forms exhibit good neuroprotective activity. They are therefore a potential source of raw materials from the *Aurantiochytrium* sp. SC145 marine microalga of Vietnam that can be used for the production of food beneficial to human health, especially for those working long hours in stressful, high-temperature, light-intense, outdoor conditions such as soldiers, construction workers, and mineworkers.

## 3. Materials and Methods

### 3.1. Strain and Culture

In this study, we isolated the heterotrophic marine microalga *Aurantiochytrium* sp. SC145 (SC145 strain) from leaf and seagrass specimens taken from Sand Cay (Son Ca) Island, Vietnam (at 10°22′42″ N latitude–114°28′33″ E longitude), from May to June and from September to November 2021; these specimens were deposited at the Department of Algal Biotechnology, Institute of Biotechnology, Vietnam Academy of Science and Technology, Vietnam under accession number ASC145 (Figure 1).

This strain was kept on a GPY medium (0.2% glucose, 0.1% polypeptide, 0.05% yeast extract, 1.5% agar, and 17.5 g/L artificial sea water—ASW). The primary seed culture was prepared by placing colonies of the SC145 strain in a Petri dish in a 1000 mL Erlenmeyer flask with 350 mL of an M1 liquid medium containing 30 g/L glucose, 10 g/L yeast extract, and 2.5% NaCl.

The primary seed culture flasks were incubated for 96 h at 28–30 °C with shaking at 250 rpm. The highest levels of the dry biomass and lipid content were 12.39 ± 1.62 g/L and 27.82 ± 1.67% dry cell weight (DCW), respectively.

The size of the inoculum was 2% of the total liquid volume in the fermentor. In the 30 L fermentor, the SC145 strain grew well in a medium containing 9% glucose and 1% industrial yeast extract. The temperature was kept at 28–30 °C, and the dissolved oxygen level was maintained above 10% by manually increasing the stirring speed from 350 rpm to a maximum of 450 rpm. The aeration rate was maintained at 0.5 volume air/(vol. medium)/min using a 0.2 mm filter. Instead of antifoam, 50 mL Neptune^®^ Gold vegetable oil (made by Cai Lan Vegetable Oil Limited Company, Ha Long City, Quang Ninh Province, Vietnam) was added to the 30 L fermentor.

The SC145 strain biomass was harvested after 120 h of fermentation. The culture was conducted in a nutrient medium containing 9% glucose and 1% industrial yeast extract of 3% salinity at room temperature (from 28 to 30 °C) in a 30 L fermentor (handmade). The growth of the *Aurantiochytrium* sp. SC145 species reached its maximum after 120 h of culture, with dry biomass and lipid content reaching 31.183 ± 2.630 g/L and 25.292 ± 1.430% of DCW, respectively. The fatty acid profile was mainly dominated by C16:0 (palmitic acid; 1.288% of DCW), C18:0 (stearic acid; 0.738% of DCW), C18:1 ω-9 (oleic acid; 2.134% of DCW), C18:2ω-6 (linoleic acid; 0.016% of DCW), C18:3 ω-3 (linolenic acid; 1.076% of DCW), C20:0 (arachidic acid; 0.178% of DCW), C20:5ω-3 (EPA; 0.051% of DCW), C22:6 ω-3 (DHA; 0.129% of DCW), total fatty acid (TFA; 6. 655% of DCW), saturated fatty acid (SFA; 2.566% of DCW), monounsaturated fatty acid (MUFA; 2.193% of DCW), and polyunsaturated fatty acids (PUFAs; 1.318% of DCW).

**Figure 5:**
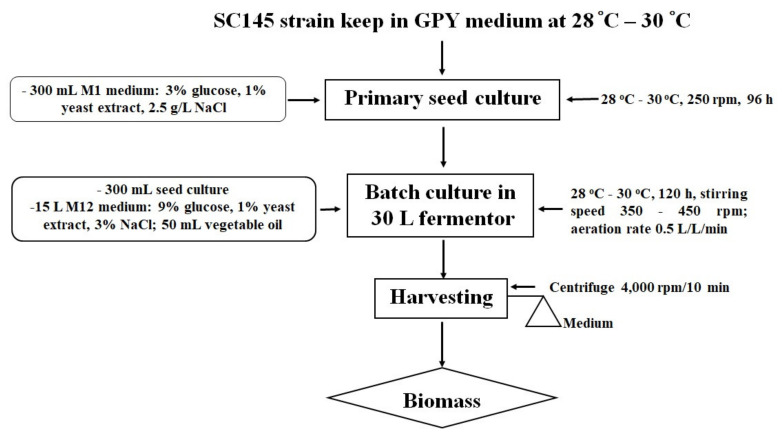
The procedure for cultivation of *Aurantiochytrium* sp. SC145 for the exploitation of PUFAs in the FAAE and FFA forms.

Algal biomass was harvested at the early stationary phase in the growth curve after centrifugation at 4000 rpm for 10 min. The algal paste was washed three times with sterile distilled water and then dried to a constant weight in a drying oven at 50 °C and stored in desiccators. The procedure followed for cultivation of *Aurantiochytrium* sp. SC145 for the exploitation of secondary metabolites is shown in Figure 5.

### 3.2. Determination of Microalgal Growth

For the determination of algal growth in terms of DCW, a 10 mL culture broth after 120 h of culture (in the early stationary phase of the growth curve) was collected and centrifuged to obtain the biomass. After centrifugation, the cell biomass was transferred to a cup with an identified weight mass and dried at 105 °C to a constant weight, three consecutive times, as described in a report by Dang et al. [62]. The DCW of each sample was determined according to the formula:

DCW (g) = Weight (cup + algal biomass) − Weight (cup)

### 3.3. Morphological Observation

The *Aurantiochytrium* sp. SC145 strain was maintained in a slant culture with a GPY⁄agar medium at 28 °C. Living cells in the colony on the agar plate and the M1 liquid medium were observed with Olympus CX21 and BX51 light microscopes and a JSM-5410L scanning electron microscope (JEOL Company, Tokyo, Japan) [62].

### 3.4. Transmission Electron Microscopy (TEM)

After cultivation, the cell suspension was centrifuged at 3000× *g* for 5 min. The cells were fixed in 2.5% glutaraldehyde in a 0.1 M cacodylate buffer (pH 7.2) and kept overnight at 4 °C. The sample was then carefully rinsed with a 0.1 M cacodylate buffer (pH 7.2) several times. The final cells were fixed with 1% OsO_4_ (osmium tetroxide) in the same buffer for 60 min, rinsed carefully with a 0.1 M cacodylate buffer (pH 7.2) several times, dehydrated in graded ethanol (from 50 to 100%), washed in propylene oxide, and infiltrated for 6 h in a 1:1 mixture of propylene oxide and epoxydic resin. The cells were then embedded in the resin. Thin sections were obtained with an ultramicrotome (Ultracut UC6; Leica Microsystems GmbH, Wetzlar, Germany) and stained with uranyl acetate and lead citrate. The ultrathin sections were then observed with a transmission electron microscope operating at 80 kV [63].

### 3.5. Scanning Electron Microscopy (SEM)

The specimens were fixed with 2.5% glutaraldehyde in 0.1 M cacodylate at pH 7.2 for 1 h or overnight. Then, they were washed three times in 0.1 M cacodylate, followed by post-fixing treatment in 0.1 M cacodylate with 1% OsO_4_ for 20 min. The samples were then washed three times in 0.1 M cacodylate and dehydrated with 50%, 70%, 90%, and 100% ethanol. After dehydration, the samples underwent critical point drying, and were mounted onto metal stubs then sputter-coated with gold and an ion coater. The prepared samples were examined with an FE-SEM S4800 scanning electron microscope [64].

### 3.6. Phylogenetic Analysis

The scientific name of the marine microalgae strain of *Aurantiochytrium* sp. SC145 was identified by comparing the nucleotide sequences of a partial 18S rRNA gene. The specific FA1/RA1 primer pair was utilized to amplify the partial 18S rRNA gene of the marine microalgae genera of *Aurantiochytrium, Schizochytrium,* and *Thraustochytrium* with a predicted size of 650 bps and sequences of FA1: 5′-AAAGATTAAGCCATGCATGT-3′ and RA1: 5′-AGCTTTTTAACTGCAACAAC-3′, as published by Mo et al. [65]. The PCR reaction mixture had a total volume of 20 μL, consisting of 2 μL of a green buffer (2X, Thermo Fisher Scientific, Waltham, MA, USA), 1 μL of each primer (10 pmol/μL), 2 μL of a DNA template (50–100 ng/μL), 0.5 μL Taq polymerase (2 u/μL), 1.5 μL dNTP, 0.5 μL MgCl_2_ (50 mM), and 11.5 μL deionized water. PCR amplification was performed with a Veriti^®^ 96-well thermal cycler (Applied Biosystems^®^, Life Technology, Thermo Fisher Scientific, Waltham, MA, USA) in a cycling program of denaturation for 3 min at 95 °C and 35 amplification cycles of 1 min at 94 °C, 1 min at 50 °C, and 1 min at 72 °C, with a 10 min extended elongation step. PCR production was visualized on an ethidium bromide-stained 1.2% agarose gel. The 18S rRNA gene sequences of species belonging to the genera *Aurantiochytrium, Schizochytrium, Thraustochytrium,* and *Monohizochytrium,* and species belonging to the genus *Diplophylus* registered in the GeneBank database were used as outgroups to construct the phylogenetic tree [66]. PCR products were precipitated with ethanol and amplified with a BigDye Terminator v3.1 cycle sequencing kit (Thermo Fisher Scientific, Waltham, MA, USA) according to the manufacturer’s recommendations. Sequence analysis was conducted using standard nucleotide BLAST, blastn (https://blast.ncbi.nlm.nih.gov/ (accessed on 21 July 2022)), a nucleotide collection (nr/nt) database, and ClustalX (1.81) and MEGA X software [67].

### 3.7. Methods for Determining Optimal Culture Conditions in Erlenmeyer Flask

In order to determine the optimal culture conditions for the SC145 strain in a 250 mL Erlenmeyer flask, the effects on algal growth of Bajpai, M1, and GPY nutritional media; glucose, maltose, maltodextrin, and starch carbon sources; 1, 3, 5, 7, and 9% glucose levels; yeast extract, (NH4)_2_SO_4_, CH_3_COONH_4_, and NaNO_3_ nitrogen sources; 0.5, 1, 1.5, and 2% concentrations of yeast extract; and temperatures of 15, 20, 25, 28, 30, and 35 °C were studied. After 120 h of cultivation, samples were taken to evaluate the parameters of cell density, DCW, and lipid content.

### 3.8. Methods for Determining Optimal Culture Conditions in a 30 L Fermentor

To determine the optimal culture conditions of the SC145 strain in a 30 L fermentor, the effects on algal growth of glucose concentrations (3, 6, 9, and 12%) and nitrogen sources (yeast extract and industrial yeast extract) were studied. After 120 h of cultivation, samples were taken for evaluation of the parameters of cell density, DCW, and lipid content.

### 3.9. Extraction and Determination of Total Carotenoid and Astaxanthin

The total carotenoid content was determined as described by Furlan et al. [68]. First, 1 g of fresh biomass was ground using 10 mL 90% acetone with a mortar and pestle. This mixture was transferred to tubes enveloped by silver foil. After washing the porcelain mortar and pestle, the second step was repeated. The samples were kept in the cold for at least 2 h, and after extraction or centrifugation at 10,000 rpm at 4 °C for 10 min the mixture was then filtered using filter paper to obtain the supernatant. Then, 10 mL of this solution was poured into a 90% acetone solution and placed in a bottle of penicillin. The final solution was light yellow. Note that the extraction was carried out in the dark and cold. The solution was studied using a spectrophotometer to measure the absorbance (OD) at wavelengths of 477 nm and 480 nm.

The total carotenoid content (μg/g of DCW) was calculated with the formula:

$$\mathrm{Total}\;\mathrm{carotenoid}\;\mathrm{content}=\frac{A477\mathrm{nm}\;.\;\mathrm{Vextrac}\;.\;\mathrm{DF}.\;.}{0.2\;\times \;\mathrm{Wsample}}$$

where A477 nm is the optical density absorbed at a wavelength of 477 nm, V_extract_ is the extract volume (mL), DF is the dilution factor (final volume divided by the initial volume), 0.2 is the value of the 1 μg/mL carotenoid solution measured at A477 nm, and Wsample is the sample weight (g).

The astaxanthin concentration (mg/L) was calculated with the formula [69]:

Astaxanthin concentration (mg/L) = 4.5 × A_480_ × (Va/Vb) × f

where A480 nm is the optical density absorbed at a wavelength of 480 nm, Va (mL) is the volume of the solvent, Vb (mL) is the volume of the algal sample, and f is the dilution ratio.

### 3.10. Extraction and Determination of Total Protein and Carbohydrate Contents

The total protein content was determined according to the method described by Cakmak et al. [70] and Bradford [71]. The carbohydrate content was determined according to the method described by Sun et al. [72].

### 3.11. Analysis of Fatty Acid Composition of Microalgal Biomass

The fatty acid composition of the microalgal biomass (in terms of PUFAs in the alkyl ester and free forms) was determined according to the LFOD-TST-8444 (GC–FID) method conducted at SGS Vietnam Ltd. The fatty acid composition and content were analyzed using an HP-6890 gas chromatograph coupled with an HP-5MS Agilent 5973 mass selective detector column (0.25 m × 30 m × 0.25 mm); the carrier gas was He and the temperature program was 80 °C for 1 min, increased to 150 °C at a rate of 4 °C/min, then increased to 260 °C and held for 10 min at a rate of 10 °C/min. The WILEY275. L and NIST 98. L mass spectrum libraries were used according to the German ISO/FDIS 5590:1998 standard [73].

### 3.12. PUFAs Rich in Omega 3–6 Fatty Acids in the Free Form (FFA)

The total lipid extraction was carried out as described by Bligh and Dyer [74] with some modifications described by Dang et al. [2]. Twenty grams of dried biomass was ground using a porcelain mortar and pestle with distilled water (at 10% of the total volume or available water in fresh biomass) for 45 min. Then, the mixture was placed into a Pyrex bottle and n-hexane was added at a 1:10 (*w*:*v*) ratio of biomass–n-hexane. The mixture was continuously stirred at 70 °C for 3 h. After the reaction, the mixture was allowed to cool to room temperature, then centrifuged at 3000 rpm for 5 min in order to collect the total lipids in the upper layer. Oil was obtained following the removal of the n-hexane.

Ten grams of total lipids were added to 1.8 g of NaOH, 4.4 mL of distilled water, and 21 mL of ethanol. The mixture was heated to 70 °C and continuously stirred for 3 h. After finishing this step, the mixture was added to 20 mL of 3% NaCl and centrifuged at 6000 rpm for 40 min at room temperature to remove the unsaponifiable fraction. The saponification fraction was added to a solution of HCl until the pH reached 2, and then centrifuged at 6000 rpm for 40 min. Then, n-hexane was added to the solution in order to obtain free fatty acids (FFAs). FFAs were collected after the removal of n-hexane using a rotary evaporator at 70 °C.

The obtained FFAs were then added to a mixture of urea and ethanol at the FFA–urea–ethanol ratio of 1:4:36 (*w*:*w*:*v*). The mixture was completely dissolved in a magnetic stirrer at 60 °C for 10 min and then kept at 4 °C for 15 h. After the complexing process, the mixture was divided into two phases—urea and non-urea complexing—and separated with filter paper. The non-urea complexing phase was completely removed using ethanol and washed with warm water (at 60 °C) to eliminate redundant urea. Then, the mixture was added to a mixture of n-hexane and omega 3–6 PUFAs; FFAs were collected after removing n-hexane with a vacuum rotary evaporator at 70 °C.

### 3.13. PUFAs Rich in Omega 3–6 Fatty Acids in the Alkyl Ester Form (FAAE)

Twenty grams of dried biomass was methylated in a mixture of 10% HCl in methanol and dichlomethane at a biomass–methanol–dichlomethane ratio of 1:10:5 (*w*:*v*:*v*). The mixture was heated at 70 °C for 3 h and then mixed well. After the reaction, the mixture was allowed to cool to room temperature. Then, the mixture was added to a 40 mL solution of saturated NaCl and n-hexane. The mixture was shaken and the n-hexane phase containing the TFA was collected. The TFA content was obtained after removing the n-hexane with a rotary evaporator. The obtained TFA sample was added to a mixture of urea and methanol at a TFA–urea–methanol ratio of 1:4:20 (*w*:*w*:*v*). The mixture was completely dissolved in a magnetic stirrer, heated at 60 °C for 10 min, and complexed at 4 °C for 15 h. After the complexing process, the mixture was divided into two phases consisting of urea and non-urea complexing, before being separated using filter paper. The non-urea complexing phase was completely removed with methanol, and washed with warm water (at 60 °C) to eliminate the redundant urea. Then, n-hexane was added to the mixture to dissolve the omega 3–6 fatty acids. Finally, n-hexane was evaporated with a vacuum rotary evaporator at 70 °C to obtain the omega 3–6 PUFA mixture in the alkyl ester form (FAAEs) [75].

### 3.14. Extraction and Purification of ALA, LA, EPA and DHA

The ALA, LA, EPA, and DHA polyunsaturated fatty acids were extracted and purified as described by Hang et al. [76]. The structures of purified compounds were confirmed with nuclear magnetic resonance (NMR) spectroscopy. NMR experiments were performed using a Bruker Avance e 500 MHz spectrometer (Bruker, Karlsruhe, Germany) at operating frequencies of 500 MHz (^1^H-NMR), at the Center for Apply Spectroscopy, Institute of Chemistry, Vietnam Academy of Science and Technology, Hanoi, Vietnam.

Docosahexaenoic acid (DHA): Yellow oil. ^1^H NMR (500 MHz, CDCl_3_, δ, ppm, J/Hz): 5.41–5.36 (12H, m, H-4,5, 7, 8, 10, 11, 13, 14 16, 17, 19, 20), 2.84–2.80 (10H, m, H-6, 9, 12, 15, 18), 2.41 (4H, m, H-2, 3), 2.08 (2H, m, H-21), 0.97(3H, t, J = 7.5, H-22) [77].

Eicosapentaenoic acid (EPA): Colorless oil. ^1^H-NMR (500 MHz, CDCl_3_, δ, ppm, J/Hz): 0.97 (3H, t, J = 7.7; H-20), 1.66 (2H, m, H-3), 2.08 (2H, m, H-19), 2.13 (2H, m, H-4), 2.29 (2H, t, J = 7.3; H-2), 2.86–2.81 (8H, m, H-7, 10, 13, 16), 5.40–5.30 (10H, m, H-5, 6, 8, 9, 11, 12, 14, 15, 17, 18) [78].

Alpha-linolenic acid (ALA): Colorless oil ^1^H NMR (500 MHz, CDCl_3_, δ ppm, J/Hz): 11.4 (1H, br. s, -COOH), 5.3 ~ 5.4 (m, 6H, H-9, 10, 12, 13, 15, 16), 2.8 (4H, t, H-11, 14), 2.35 (2H, t, H-2), 2.06 (4H, m, H-8, 17), 1.63 (2H, m, H-3), 1.32 (8H, m, H-4, 5, 6, 7), 0.98 (3H, t, H-8) [79].

Linoleic acid (LA): Colorless oil; ^1^H-NMR (500 MHz, CDCl3, δ ppm, J/Hz): 5.50–5.30 (4H, m, H-9, 10, 12, 13), 2.77 (2H, t, J = 6,5 Hz, H-11), 2.30 (2H, t, J = 7.5 Hz, H-2), 2.06–2.02 (4H, q, J = 7.0 Hz, H-8, 14), 1.62 (2H, m), 1.37–1.25 (14H, m), 0.89 (3H, t, J = 7,0 Hz, H-18) [78].

### 3.15. Acute Oral Toxicity Study

Acute toxicity and LD_50_ (50% lethal dose) values were evaluated as described by the guidelines of the World Health Organization [80] and the Organization for Economic Co-operation and Development (OECD) [81]. Ninety-six white Swiss mice (male and female, weights ranging from 18 to 22 g) were randomly divided into 12 groups with 8 mice per group and treated with PUFAs rich in omega 3–6 fatty acids in the form of FFA or FAAE at different dosages (3.82, 6.11, 8.40, 10.69, 12.98, and 15.35 g/kg body weight/day), as described previously [15]. Mortality or signs of morbidity in mice in each group were recorded over 72 h. Then, animals were continuously monitored until the end of the 7th day after the first oral administration.

### 3.16. Subchronic Oral Toxicity Study

Subchronic toxicity was evaluated as described in the guidelines of the WHO [56] and OECD [81]. Forty white Wistar rats (male and female, weights ranging from 160 to 180 g) were randomly divided into 5 groups with 8 rats per group. Groups 1 and 2 were exposed to doses of 78.5 and 353.3 mg/kg/day of FAAEs, respectively. Groups 3 and 4 were exposed to doses of 78.5 and 353.3 mg/kg/day of FFA, respectively. An untreated group (control group) received normal saline at a dosage of 1.00 mL/kg/day. All experimental and control groups were evaluated over 90 days. The hematological parameters, biochemical parameters, and liver and kidney functions of the rats were analyzed before (D0) and after oral administration for 45 days (D45) and 90 days (D90), as described by Thom and Hong [15].

### 3.17. DPPH Assay

The 2,2-diphenyl-1-picrylhydrazyl (DPPH) assay was used as described by Alam et al. [82]. Firstly, PUFAs in the FAAE and FFA forms, LA, ALA, EPA, and DHA were prepared in dimethyl sulfoxide (DMSO). DMSO was also used as a control group. Ascorbic acid, a potent reducing and antioxidant agent, was used as a positive control. A DPPH solution was dissolved in methanol to a concentration of 0.5 mM. Then, 100 μL of each sample or DMSO was added to a 100 μL DPPH solution and left to stand for 30 min in the dark at room temperature. After incubation, the absorbance of the reaction mixtures was measured at an absorption wavelength of 517 nm using a microplate reader (Thermo Fisher Scientific, Inc., Waltham, MA, USA). The percentage of the scavenging effect on the DPPH inhibition was calculated with equation:

% Inhibition of DPPH radical = [(A_0_ − A_1_)/A_0_] × 100

where A_0_ is the absorbance of the control and A1 is the absorbance of the samples. All experiments were performed in triplicate. The results are given as IC_50_ corresponding to the concentration achieving 50% inhibition.

### 3.18. AChE Inhibitory Activity Assay

The AChE inhibitory activities of PUFAs in the FAAE and FFA forms, LA, ALA, EPA and DHA were evaluated using an acetylcholinesterase inhibitor screening kit (Sigma, St. Louis, MO, USA) according to the provided instructions. Galantamine, which is used for treatment of Alzheimer’s disease, was used as a positive control. Absorbance was measured at 412 nm with a microplate reader (Thermo Fisher Scientific, Inc., Waltham, MA, USA). All experiments were performed in triplicate. The results are given as IC_50_. The percentage inhibition of AChE activity was calculated as follows:

% Inhibition = (1 – ΔA_Test Cpd_/ΔA_No Inhibitor_) × 100%

where ΔA_Test Cpd_ is the A_412_ value of a sample well at 0 min subtracted from the A_412_ value of the same well at 10 min, and ΔA_No Inhibitor_ is the A_412_ value of the No Inhibitor control well at 0 min subtracted from the A_412_ value of the No Inhibitor control well at 10 min.

### 3.19. Cell Culture and Treatment

C6 rat glial cells (ATCC, CCL-107™) were obtained from Dr. Duong Hoang Nguyen, Center for Soft Matter and Biological Physics, Center for High Technology Development, Vietnam Academy of Science and Technology. The cells were cultured in high-glucose Dulbecco’s Minimum Essential Medium (DMEM) supplemented with 10% (*v*/*v*) fetal bovine serum (FBS) and 1% (*v*/*v*) penicillin/streptomycin under 5% CO_2_ at 37 °C.

For assessment of the protective ability of PUFAs in the FAAE and FFA forms, EPA, DHA, LA, and ALA against oxidative stress caused by H_2_O_2_, C6 cells were cultured in high-glucose DMEM in 96-well culture plates at a density of 0.5 × 10^5^ cells/well for 24 h. Then, the cells were incubated with PUFAs in the FAAE and FFA forms (0.4, 2 and 10 µg/mL), EPA, DHA, LA, ALA (0.4, 2, 10 μM), or ascorbic acid (20 µg/mL) as a positive control for another 24 h, followed by 1 h of incubation with a H_2_O_2_ solution (10 μM). The samples’ cytoprotective effects against oxidative stress induced by H_2_O_2_ on C6 cells were assessed in terms of the cell survival rate using a 3-(4,5-dimethylthiazol-2-yl)-2,5-diphenyltetrazolium bromide (MTT) assay.

For the assessment of neuroprotection against Aβ_25-35_-induced cytotoxicity in the C6 cell line provided by PUFAs in the FAAE and FFA forms, EPA, DHA, LA, and ALA, cells were cultured for 24 h in high-glucose DMEM in 96-well culture plates at a density of 0.5 × 10^5^ cells/well. Then, the cells were incubated with PUFAs in the FAAE and FFA forms (0.4, 2 and 10 µg/mL), EPA, DHA, LA, ALA (0.4, 2 and 10 µM) or galantamine (1 µg/mL; Sopharma AD, Sofia, Bulgaria) as a positive control for another 24 h, followed by 24 h of incubation with an Aβ_25-35_ protein (20 μM) [56]. Cell viability was determined using the MTT assay method.

### 3.20. MTT Assay

After treatment, C6 cells were incubated with 5 μL MTT solution (5 mg/mL) at 37 °C and 5% CO_2_ for 4 h. After 4 h of incubation, the media were removed and purple MTT formazan crystals were dissolved with the addition of 100 μL of DMSO to each well. The absorbance was measured at 570 nm with a microplate reader (Thermo Fisher Scientific, Inc., Waltham, MA, USA). In this experiment, each concentration was assessed in triplicate. The cell survival rate was calculated as follows:

Cell survival rate (%) = (A_1_/A_0_) × 100

where A_0_ is the absorbance of control and A_1_ is the absorbance of the samples. All experiments were performed in triplicate.

### 3.21. Statistics Analysis

All experiments were performed in triplicate. Data are expressed as the mean ± standard error of the mean (SEM). Statistically significant differences for control-group comparisons were evaluated using Student’s *t* test. Differences were considered statistically significant at *p* < 0.05. Experimental data were processed using Excel software and statistically analyzed via one-way ANOVA and Duncan’s post hoc test at a significance level of *p* < 0.05.

## 4. Conclusions

From samples collected in May–June 2021 in the coastal area of Sand Cay (Son Ca) Island, Vietnam, *Aurantiochytrium* sp. SC145 heterotrophic marine microalga (capable of producing high levels of PUFAs and other components) was isolated and stably cultured under laboratory conditions. The most suitable conditions for the biomass cultivation of the SC145 strain for use as a raw material for PUFA extraction were found to be the M1 medium containing 3% glucose and 1% yeast extract at a temperature of 28–30 °C on the flask scale, and the M12 medium containing 9% glucose and 1% industrial yeast extract on the 30 L fermentor scale. In the 30 L fermentor, the dry cell weight, lipid content, protein content, and carbohydrate content reached their highest values of 31.18 ± 2.63 g/L, 25.29 ± 1.43%, 7.93 ± 0.17%, and 15.21 ± 0.02% of DCW, respectively. In addition, carotenoid and astaxanthin contents reached 143.67 ± 0.35 and 8.07 ± 0.17 µg/L, respectively. The fatty acid composition of the SC145 strain biomass mainly comprised omega 3–6–9 fatty acids, which accounted for 51.60% of TFA. The PUFAs extracted from the dry biomass of the *Aurantiochytrium* sp. SC145 strain in the free and alkyl ester fatty acid forms rich in omega 3–6–9 fatty acids (with main constituents of EPA, DHA, and ALA) showed antioxidant and AChE inhibitory properties and neuroprotective activity against H_2_O_2_- and amyloid-ß protein fragment 25–35 (Aβ_25-35_)-induced damage in C6 cells. The mixtures of PUFAs in the FFA and FAAE forms were found to be safe according to acute and subchronic oral toxicity tests in the animal model. This study on PUFAs rich in omega-3–6–9 acids present in the *Aurantiochytrium* sp. SC145 strain contributes to the general understanding of the exploitation and development of drugs and functional foods based on marine microalgae. These PUFAs were also shown to be promising ingredients for the prevention and treatment of Alzheimer’s disease.

The neuroprotective mechanism of PUFA mixtures in the FFA or FAAE forms (including the main effects caused by the individual fatty acids ALA, EPA, and DHA) requires further elucidation in future studies.

## Figures and Tables

**Table 1:** The effects of environmental parameters on the cell density, DCW, and lipid content of *Aurantiochytrium* sp. SC145 in an Erlenmeyer flask after 120 h of culture.

Culture Conditions		Cell Density (×10^6^ Cells/mL)	DCW (g/L)	Lipid (% DCW)
Nutritional media	Bajpai	45.48 ± 1.23 ^b^	7.13 ± 0.31 ^b^	17.32 ± 1.37 ^b^
	M1	120.17 ± 2.76 ^a^	12.27 ± 0.53 ^a^	27.41 ± 0.73 ^a^
	GPY	10.20 ± 1.01 ^c^	2.25 ± 0.05 ^c^	4.25 ± 1.06 ^c^
Temperatures (°C)	15	45.13 ± 0.51 ^f^	6.28 ± 0.31 ^c^	8.12 ± 0.43 ^c^
	20	96.15 ± 1.04 ^d^	9.89 ± 1.02 ^b^	21.65 ± 1.63 ^b^
	25	117.43 ± 1.89 ^b^	12.10 ± 1.33 ^a^	27.84 ± 1.27 ^a^
	28	123.68 ± 2.73 ^a^	12.39 ± 1.62 ^a^	27.82 ± 1.67 ^a^
	30	112.71 ± 1.22 ^c^	8.03 ± 0.82 ^c^	20.56 ± 0.86 ^b^
	35	85.35 ± 0.67 ^e^	4.29 ± 0.37 ^d^	7.19 ± 0.31 ^c^
Carbon sources	Maltose	32.37 ± 0.61 ^c^	3.95 ± 0.04 ^c^	10.74 ± 1.16 ^c^
	Glucose	121.48 ± 1.06 ^a^	12.79 ± 1.15 ^a^	26.63 ± 1.18 ^a^
	Starch	30.67 ± 0.19 ^d^	3.73 ± 0.13 ^d^	4.61 ± 0.76 ^d^
	Maltodextrin	73.42 ± 0.53 ^b^	8.78 ± 1.11 ^b^	15.37 ± 1.03 ^b^
Concentrations of glucose (%)	1	53.29 ± 0.62 ^d^	5.09 ± 0.12 ^b^	16.13 ± 1.21 ^c^
	3	127.62 ± 2.03 ^a^	12.69 ± 1.12 ^a^	26.76 ± 0.24 ^a^
	5	121.15 ± 1.65 ^b^	11.73 ± 1.17 ^a^	24.37 ± 0.25 ^b^
	7	118.57 ± 1.18 ^bc^	11.78 ± 0.64 ^a^	23.85 ± 0.36 ^b^
	9	117.02 ± 1.43 ^c^	11.21 ± 0.21 ^a^	23.51 ± 0.51 ^b^
Nitrogen sources	(NH4)_2_SO_4_	68.03 ± 0.88 ^d^	6.32 ± 0.78 ^d^	10.37 ± 0.72 ^c^
	CH_3_COONH_4_	86.69 ± 1.71 ^c^	9.53 ± 0.34 ^c^	21.69 ± 0.30 ^b^
	Yeast extract	125.48 ± 1.89 ^a^	12.36 ± 0.75 ^a^	28.32 ± 1.54 ^a^
	NaNO_3_	114.37 ± 1.34 ^b^	10.82 ± 0.51 ^b^	20.87 ± 1.29 ^b^
Concentrations of yeast extract (%)	0.5	8.15 ± 0.41 ^d^	8.77 ± 0.31 ^b^	22.14 ± 0.15 ^c^
	1.0	128.47 ± 1.08 ^a^	12.85 ± 1.37 ^a^	28.34 ± 1.21 ^a^
	1.5	119.93 ± 1.38 ^b^	12.18 ± 0.23 ^a^	28.02 ± 1.43 ^a^
	2.0	97.34 ± 0.56 ^c^	9.37 ± 0.64 ^b^	23.52 ± 0.76 ^b^

Note: Different superscript letters (a, b, c, d, and e) indicate a statistically significant difference in the sample mean (one-way ANOVA with Duncan’s post hoc test, *p* < 0.05).

**Table 2:** The effects of environmental parameters on cell density, DCW, and lipid content of *Aurantiochytrium* sp. SC145 after 120 h of culture in a 30 L fermentor.

Culture Conditions		Cell Density (×10^6^ Cells/mL)	DCW (g/L)	Lipid (% DCW)
Concentration of glucose (%)	3	115.43 ± 2.48 ^d^	22.36 ± 1.45 ^c^	16.34 ± 0.15 ^d^
	6	132.12 ± 2.04 ^c^	28.82 ± 1.02 ^b^	22.01 ± 0.26 ^c^
	9	154.75 ± 1.14 ^a^	31.15 ± 1.13 ^a^	24.92 ± 0.31 ^a^
	12	147.02 ± 1.10 ^b^	31.12 ± 0.18 ^a^	24.11 ± 0.43 ^b^
Nitrogen source	Industrial yeast extract	148.51 ± 0.67	31.18 ± 2.63	25.29 ± 1.43
	Pure yeast extract	145.27 ± 1.83	31.89 ± 1.17	26.15 ± 1.56

Note: Different superscript letters (a, b, c, and d) indicate a statistically significant difference in the sample mean (one-way ANOVA with Duncan’s post hoc test, *p* < 0.05).

**Table 3:** Contents of lipids, proteins, carbohydrates, and carotenoids in the SC145 strain.

Parameter	Content
Lipid (% DCW)	25.29 ± 1.43
Protein (% DCW)	7.93 ± 0.17
Carbohydrate (% DCW)	15.21 ± 0.02
Carotenoid (µg/L)	143.67 ± 0.35
Astaxanthin (µg/L)	8.07 ± 0.17

**Table 4:** Fatty acid composition of the dry biomass of the SC145 strain cultured in a 30 L fermentation system after 120 h of culture.

No	Fatty Acid Composition	Fatty Acid Content (g/100 g DCW)	No	Fatty Acid Composition	Fatty Acid Content (g/100 g DCW)
1	C4:0 Butyric acid	0.000	28	C20:1 Gondoic acid	0.019
2	C6:0 Caproic acid	0.002	29	C20:2 Eicosadienoic acid	0.009
3	C8:0 Capryllic acid	0.003	30	C20:3 gamma -Eicosatrienoic acid	0.000
4	C10:0 Capric acid	0.000	31	C20:3 Eicosatrienoic acid	0.000
5	C11:0 Undecanoic acid	0.000	32	C20:4 Arachidonic acid (ARA)	0.000
6	C12:0 Lauric acid	0.003	33	C20:5 Eicosapentaenoic acid (EPA)	0.051
7	C13:0 Tridecanoic acid	0.000	34	C21:0 Heneicosanoic acid	0.000
8	C14:0 Myristic acid	0.016	35	C22:0 Behenic acid	0.147
9	C14:1 Myristoleic acid	0.000	36	C22:1 Erucic acid	0.003
10	C15:0 Pentadecanoic acid	0.008	37	C22:2 Docosadienoic acid	0.000
11	C15:1 Pentadecenoic acid	0.000	38	C22:4 Docosatetraenoic acid	0.000
12	C16:0 Palmitic acid	1.288	39	C22:5 all cis-4, 7, 10, 13, 16	0.000
13	C16:1 cis-7-Hexadecenoic acid	0.001	40	C22:5 all cis-7, 10, 13, 16	0.000
14	C16:1 Palmitoleic acid	0.037	41	C22:6 Docosahexaenoic acid	0.129
15	C17:0 Heptadecanoic acid	0.058	42	C23:0 Tricosanoic acid	0.005
16	C17:1 10-Heptadecenoic acid	0.000	43	C24:0 Lignoceric acid	0.120
17	C18:0 Stearic acid	0.738	44	C24:1 Nevonic acid	0.000
18	C18:1 Tran fatty acids	0.028	45	Other fatty acids	0.549
19	C18:1 ω-9 Oleic acid	2.134	46	Total fatty acids	6.655
20	C18:2 Trans fatty acids	0.025	47	Total fat (as triglyceride fat)	6.758
21	C18:2 Rumenic acid (CLA)	0.000	48	Saturated fatty acids	2.566
22	C18:2 ω-6 Linoleic acid (LA)	0.016	49	Trans fatty acids	0.060
23	C18:3 Trans fatty acids	0.007	50	Monounsaturated fatty acids	2.193
24	C18:3 ω-3 Linolenic acid (ALA)	1.076	51	Polyunsaturated fatty acids	1.318
25	C18:3 gamma-Linolenic acid	0.000	52	Omega-3	1.261
26	C18:4 Octadecatetraenoic acid	0.005	53	Omega-6	0.025
27	C20:0 Arachidic acid	0.178	54	Omega-9	2.157

**Table 5:** Fatty acid composition in PUFAs mixture after urea complexation (fatty acid in the FFA and FAAE forms).

No	Acid Name	Scientific Name	Common Name	PUFAs Contents (% TFA)	
				In FFAs Form	In FAAEs Form
1	14:0	Tetradecanoic acid	Myristic	1.38 ± 0.01	1.36 ± 0.01
2	15:0	Pentadecanoic acid		1.31 ± 0.10	0.92 ± 0.01
3	15:1 ω-5	Hexadecanoic acid		2.44 ± 0.05	2.45 ± 0.02
4	16:0	Hexandecanoic acid	Palmitic	22.93 ± 0.22	22.26 ± 0.14
5	16:1 ω-7	9-hexadecenoic acid	Palmitoleic	2.97 ± 0.02	0.71 ± 0.01
6	17:0	Heptadecanoic acid	Margnic	1.04 ± 0.01	0.88 ± 0.02
7	18:0	Octadecanoic acid	Stearic	3.75 ± 0.01	4.66 ± 0.01
8	18:1 ω-9	Cis 9- octadecenoic acid	Oleic	4.57 ± 0.02	4.32 ± 0.01
9	18:1 ω-7	11- octadecenoic acid		19.28 ± 0.06	17.74 ± 0.05
10	18:2 ω-6-t	9,12- octadecenoic acid	Linoleic (LA)	5.63 ± 0.07	5.12 ± 0.08
11	18:3 ω-6	Alpha—Linolenic acid	ALA	20.13 ± 0.01	22.02± 0.02
12	20:2 ω-6	11,14-eicosadienoic acid		2.56 ± 0.01	3.34 ± 0.01
13	20:5 ω-3	5,8,11,14,17 eicosapentaenoic acid	EPA	1.40 ± 0.02	0.36 ± 0.01
14	20:1 ω-9	11-eicosenoic acid		0.43 ± 0.02	0.24 ± 0.01
15	22:3 ω-3	11,14,17-eicosatrienoic acid		1.27 ± 0.01	1,81 ± 0.02
16	22:4 ω-6	7,10,13,16-docosatetraenoic acid		2.38 ± 0.01	2,80 ± 0.02
17	22:6 ω-3	4,7,10,13,16,19 docosahexaenoic acid	DHA	7.36 ± 0.03	8.99 ± 0.03
	Omega 3			24.53 ± 0.05	25.66 ± 0.06
	Omega 6			11.20 ± 0.10	13.76 ± 0.13
	Omega 9			5.00 ± 0.04	4.56 ± 0.02
	Total omega 3, 6, 9			40.73 ± 0.19	44.00 ± 0.21

**Table 6:** Average body-weight gain for white mice with PUFAs in the FAAE and FFA forms orally administered over 90 days.

Groups	Body Weight		
	Before Test	After 45 Days	After 90 Days
Control	178.24 ± 5.38	205.19 ± 5.39	225.89 ± 5.59
Group 1	179.26 ± 5.19	207.07 ± 4.77	227.28 ± 5.89
Group 2	179.57 ± 4.50	208.67 ± 9.18	228.51 ± 9.61
Group 3	173.43 ± 5.14	204.20 ± 5.05	219.99 ± 5.34
Group 4	175.40 ± 4.96	207.99 ± 4.56	224.33 ± 5.63

Note: Control: administered normal saline at a dosage of 1.00 mL/kg/day; groups 1 and 2: administered PUFAs in the FAAE form at dosages of 78.5 and 353.3 mg/kg/day, respectively; groups 3 and 4: administered PUFAs in the FFA form at dosages of 78.5 and 353.3 mg/kg/day, respectively.

**Table 7:** Results of hematological and biochemical parameters after 90 days of the administration of PUFAs in the FAAE and FFA forms.

Parameter	Control	Group 1	Group 2	Group 3	Group 4
***Hematological parameters***					
RBC (×10^12^ g/l)	8.57 ± 1.57	8.82 ± 2.11	8.42 ± 0.05	8.58 ± 1.57	8.42 ± 0.05
Hemoglobin (g/dL)	135.54 ± 18.58	133.24 ± 10.00	136.56 ± 13.72	135.67 ± 18.60	136.69 ±13.73
Hematocrit (%)	43.61 ± 6.17	42.37 ± 6.58	42.12 ±2.72	43.65 ± 6.18	42.16 ±2.73
Average volume off erythrocytes (fl)	52.28 ± 2.02	51.39 ± 8.12	51.51 ± 2.67	52.33 ± 2.02	51.56 ± 2.68
WBC (g/L)	11.63 ± 3.11	11.41 ± 4.68	11.42 ± 3.25	11.64 ± 3.23	11.44 ± 3.26
Platelet count (g/L)	687.36 ± 154.06	702.53 ± 249.72	638.78 ± 88.95	688.03 ± 154.21	639.40 ± 89.04
***Biological parameters in liver***					
AST (UI/L)	85.43 ± 13.42	78.03 ± 20.95	80.71 ± 11.55	85.51 ± 13.44	82.79 ± 11.56
ALT (UI/L)	71.28 ± 10.51	64.14 ± 22.58	67.71 ± 11.52	71.35 ± 10.52	70.77 ± 11.53
***Biological parameters in plasma***					
Albumin (g/L)	33.28 ± 1.44	33.66 ± 2.67	34.56 ± 2.96	33.32 ± 1.44	33.59 ± 2.96
TC (mmol/L)	1.25 ± 0.49	1.25 ± 0.45	1.22 ± 0.27	1.25 ± 0.27	1.21 ± 0.49
***Biological parameters in kidney***					
Creatinine (g/L)	64.65 ± 28.40	63.63 ± 22.58	58.27 ± 14.10	64.71 ± 28.42	62.33 ± 14.11

Note: Control: administered normal saline at a dosage of 1.00 mL/kg/day; groups 1 and 2: administered PUFAs in the FAAE form at dosages of 78.5 and 353.3 mg/kg/day, respectively; groups 3 and 4: administered PUFAs in the FFA form at dosages of 78.5 and 353.3 mg/kg/day, respectively. RBC: red blood cell; WBC: white blood cell; AST: aspartate aminotransferase; ALT: alanine aminotransferase; TC: total cholesterol.

**Table 8:** Antioxidant and neuroprotection activities of PUFAs in the FAAE and FFA forms derived from *Aurantiochytrium* sp. SC145.

Sample	DPPH Assay	AChE Inhibitory Activity
	IC_50_ (μg/mL)	IC_50_ (μg/mL)
FAAE	294.98 ± 4.03	2.65 ± 0.16
FFA	239.21 ± 5.01	4.9 ± 0.65
Ascorbic acid	19.43 ± 1.44	nd
Galantamine	nd	1.78 ± 0.15
	**IC_50_ (μM)**	**IC_50_ (μM)**
Linoleic acid (LA)	nd	nd
α-Linolenic acid (ALA)	262.54 ± 5.47	10.80 ± 0.66
Eicosapentaenoic acid (EPA)	197.87 ± 3.63	5.03 ± 0.27
*Docosahexaenoic acid (DHA)*	285.04 ± 2.71	4.40 ± 0.31

Note: nd: no determination.

## Data Availability

Not applicable.
